# Gut bacteriophage dynamics during fecal microbial transplantation in subjects with metabolic syndrome

**DOI:** 10.1080/19490976.2021.1897217

**Published:** 2021-04-01

**Authors:** Pilar Manrique, Yifan Zhu, John van der Oost, Hilde Herrema, Max Nieuwdorp, Willem M. de Vos, Mark Young

**Affiliations:** a Department of Microbiology & Immunology, Montana State University, Bozeman, MT, USA; b Laboratory of Microbiology, Wageningen University, Wageningen, The Netherlands; c Department of Internal and Vascular Medicine, Amsterdam University Medical Centers, University of Amsterdam, AZ Amsterdam, The Netherlands; d RPU Human Microbiology, University of Helsinki, Faculty of Medicine, Helsinki, Finland; e Department of Plant Sciences & Plant Pathology, Montana State University, Bozeman, MT, USA

**Keywords:** Gut microbiome bacteriophages, human gut phage metagenomics, metabolic syndrome, fecal microbial transplant

## Abstract

Metabolic Syndrome (MetS) is a growing public health concern worldwide. Individuals with MetS have an increased risk for cardiovascular (CV) disease and type 2 diabetes (T2D). These diseases – in part preventable with the treatment of MetS – increase the chances of premature death and pose a great economic burden to health systems. A healthy gut microbiota is associated with a reduction in MetS, T2D, and CV disease. Treatment of MetS with fecal microbiota transplantation (FMT) can be effective, however, its success rate is intermediate and difficult to predict. Because bacteriophages significantly affect the microbiota membership and function, the aim of this pilot study was to explore the dynamics of the gut bacteriophage community after FMT in MetS subjects. We performed a longitudinal study of stool bacteriophages from healthy donors and MetS subjects before and after FMT treatment. Subjects were assigned to either a control group (self-stool transplant, n = 3) or a treatment group (healthy-donor-stool transplant; n-recipients = 6, n-donors = 5). Stool samples were collected over an 18-week period and bacteriophage-like particles were purified and sequenced. We found that FMT from healthy donors significantly alters the gut bacteriophage community. Subjects with better clinical outcome clustered closer to the heathy donor group, suggesting that throughout the treatment, their bacteriophage community was more similar to healthy donors. Finally, we identified bacteriophage groups that could explain these differences and we examined their prevalence in individuals from a larger cohort of MetS FMT trial.

Trial information- http://www.trialregister.nl/trialreg/admin/rctview.asp?TC=2705; NTR 2705

## Introduction

MetS is a growing public health concern, currently affecting approximately one-third of people in the US and in several countries worldwide.^1–6^ Diagnosis of MetS is based on the presence of three of the following manifestations: high waist circumference, insulin resistance, high blood pressure, high plasma triglyceride levels in blood, and low high-density lipoprotein cholesterol (HDL).^7^ Obesity is an important risk factor for MetS, since approximately 75% of the obese individuals have a metabolic disorder. However, 40% of the lean individuals are also metabolically unhealthy.^7,8^ Individuals with MetS have an increased risk of developing T2D, CV disease, and nonalcoholic fatty liver disease.^1,2,7,8^ CV disease alone is the leading cause of death worldwide and, overall, individuals with MetS are four times more likely of dying from CV disease than otherwise healthy people, and twice more likely to die from premature death.^5,9^ Overall, the health cost of MetS has been estimated in the billions of dollars in the US alone, being approximately 20% higher in MetS subjects than in healthy individuals.^5,10^ Because treatment of MetS can significantly reduce the risk of developing any of these diseases, understanding MetS etiology, and developing successful treatments is needed.

The composition and structure of the gut microbial community has been associated with CV disease, obesity, diabetes, and MetS.^11^ Changes in the microbial community can affect the human host through inappropriate activation of the immune system, reduced production of beneficial metabolites for the human host, and reduced production stimulation of mammalian peptides necessary for glucose homeostasis.^11,12^ Moreover, specific bacterial species have been linked to either MetS or a healthy state.^13,14^ For instance, certain *Prevotella, Lactobacillus*, and *Bacteroides* species are associated with insulin resistance – one of the hallmarks of MetS. On the other hand, *Akkermansia municiphila* and *Faecalibacterium prausnitzii* are associated with increased insulin sensitivity.

Manipulation of the gut microbiota by fecal microbial transplantation (FMT) is a promising approach for examining the clinical effects in response to restoration of healthy gastrointestinal conditions.^15^ For example, it has been well established that the treatment of recurrent *Clostridium difficile* infections (CDI) with FTM is highly effective.^13^ However, success rate of FMT in other gastrointestinal diseases is highly variable.^16,17^ We previously showed that transplantation of healthy lean-donor fecal microbiota into MetS subjects improves peripheral insulin sensitivity based on a significant increase in the glucose disappearance rate (Rd).^14,18^ However, this effect only lasted approximately six weeks and only 65% of the subjects that received a transplant from a healthy donor showed a clinical improvement (indicated by >10% increase in glucose disappearance rate (Rd) in the first 6 weeks after treatment).^14,18^ Even though specific changes in the bacterial community composition were associated with a positive clinical outcome, it alone was not sufficient to explain the variation in patient response, underscoring the need for a greater understanding of the factors influencing MetS FMT success in order to improve clinical outcomes.^14^

Viruses of bacteria (bacteriophages, or simply phages), through bacterial predation and temporary symbiosis with specific bacterial species, are one of the main drivers of the structure and function of their host community.^19–23^ While it has been suggested that phages play a role in the success of FMT in recurrent CDI,^24^ to our knowledge, the composition and structure of the phage community in subjects with MetS and their role in MetS-FMT has not been previously investigated. We present here a temporal analysis of the phage community of adults with MetS that underwent FMT treatment with either their own stool samples (FMT control treatment group) or samples from healthy donors (FMT treatment group) (Table S1).^14^ Within the treatment group, both responders and non-responders to FMT treatment were examined. We investigated bacteriophages dynamics after treatment and attempted to determine particular features that could render MetS individuals more likely to respond to FMT treatment.

## Results

### Subjects and study design

The experimental design is summarized in Figure S1. Male, Caucasian, obese subjects with MetS were randomly assigned to one of two treatment FMT groups: control or treatment transplant (see Table S1 for subjects’ information). The control group received their own processed-stool fecal sample as a control for how the FMT procedure itself (bowel lavage followed by stool microbial community transplant) affects gut microbial community dynamics. The treatment group received processed-stool fecal sample from healthy lean donors. Subjects did not receive antibiotic treatment prior to the FMT. Based on the prior known treatment outcome (65% success rate), subjects from the treated group were further divided into Responder and Non-responder sub-groups.^14^ In this pilot study, we perform a longitudinal analysis of phage filtrates from three controls subjects (C), three non-responders (N), three responders (R), and five healthy donors (D) to investigate the bacteriophage dynamics during FMT and determine whether there were differences in the phage community based on treatment. It is important to note that stool viral-filtrates are highly enriched in bacteriophage-like particles.^25,26^ Hence, viral-like particles were isolated from stool viral-filtrates at week 0 (pre-FMT) from donors and recipients, and at weeks 3, 6, 12, and 18 post-FMT from recipients. DNA was extracted and deep-sequenced, generating a total of 46 metagenomes made up of 62 Gb of sequence data (Table S2).

### Diversity and richness of the viral community before and after FMT treatment

Sequences from phage enriched metagenomes were grouped together by individual (14-individuals’ metagenomes) and assembled to produce 28,869 putative viral sequences >3,000 bp (contigs, see Material and Methods) (Table S2). Abundance of viral sequences in each individual time point was determined by read recruitment after normalization for differences in sequencing depth (see Material and Methods). Within these 28,869 viral sequences (contigs > 3000bp), Homologous Viruses (HV) were identified and grouped together based on the Homologous Virus Diversity Index technique (HVDI).^27^ HV groups constitute sequences that likely belong to the same or highly related viruses. It is important to note that HV groups will contain viruses transferred from donors to recipients, as well as viruses shared between subjects. A total of 19,234 sequences were grouped into 1,544 HV, whereas 9,635 could not be clustered and remained as singletons. Because singletons are unique to individuals, and thus do not provide information about inter-individual similarities, analysis was focused primarily on HV groups.

Previous results demonstrated that the bacterial diversity pre-FMT (W0, before FMT) in study subjects was associated with FMT treatment outcome.^14^ Therefore, we examined the alpha-diversity of HV groups pre-FMT and post-FMT treatment. We determined phage richness (i.e. number of phage sequences) and calculated the Shannon-diversity index, which takes in account both richness and abundance of each viral sequence. Overall, there were no significant differences in viral richness pre-FMT (Richness; Donors 118 ± 55.89, Controls 162.3 ± 70.71; Treatment 113.5 ± 46.04). However, within the treatment group, the non-responders had reduced viral richness as compared to responders, which was maintained for all subjects across most sampling timepoints (Figure S2A-B). Likewise, no significant differences in Shannon-diversity index pre-FMT between treatment groups were observed (Shannon; Donors 3.7 ± 0.35, Controls 3.67 ± 0.28, Treatment 3.23 ± 0.65). Differences in viral community richness between a donor and its recipient have also been shown to be associated with recovery in CDI subjects after FMT.^24^ However, in our study, we did not detect a significant difference (NΔR: −55.33, sd: 103.6); RΔR: 44.33, sd: 29.37; *p*-value = 0.1842) (Figure S2C). Overall, these results suggest that phage richness and Shannon-diversity index are highly variable and not significantly different between healthy subjects, MetS subjects, or treatment groups.

To determine what type of viruses were present in healthy donors as compared to MetS subjects, viral sequences were taxonomically classified (Figure S3). Translated ORFs from viral contigs were compared by psiBLAST to the Phage Orthologous Groups database (POGs).^28,29^ Overall, 12,668 viral contigs (n = 28,869, 44%) were classified as bacteriophages based on a POG match. This included 268 HV contigs that were classified at the rank order or less (n = 1,544, 17%). As is common with viral sequences from the human gut, only a small fraction is similar to known bacteriophages.^28^ However, it is worth noting that the viral sequences classified as bacteriophages in this study represent a large portion of the total viral community (40% of total reads per Kb per million-RPKM for classified HV groups). Moreover, based on prior microscopy studies, the remaining sequences are likely phage sequences as well.^25,26^ In all individuals, the classified phages were dominated by members of the Caudovirales order, specifically *Siphoviridae* and *Podoviridae* families. At lower taxonomic ranks, typical taxa that has been previously associated with human gut bacteriophages were detected.^29^

### Phage transfer during FMT course

**Figure 1.**
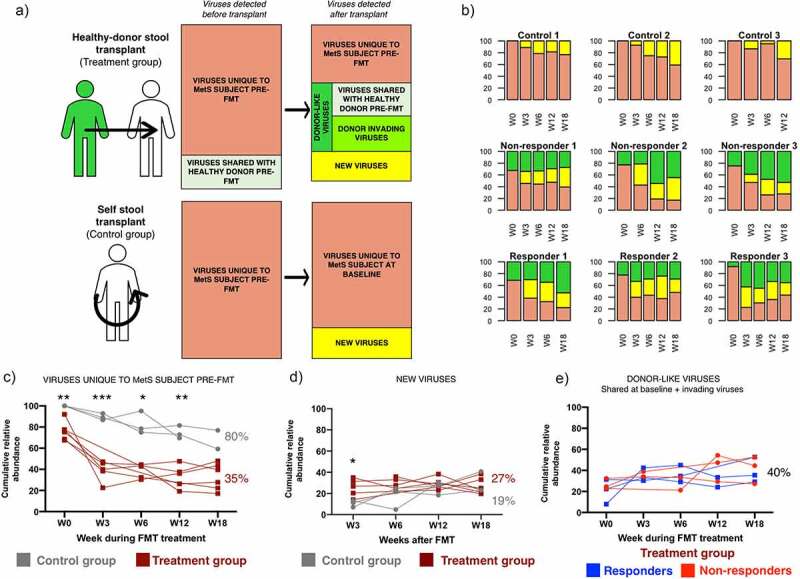
Changes in the viral community of MetS subjects after FMT. (a) Schematic representation of the composition of the viral community before and after FMT in recipients from the treatment group (top panel) and the control group (bottom panel). Individuals in the treatment group received the stool microbial community of a healthy donor (n=6). Individuals in the control group received their own stool microbial community (n=3). (b) Bar plots of the community structure after FMT in MetS subjects. Plots represent the cumulative relative abundance of the different viral categories in each individual at each timepoint (Before FMT treatment- W0 and after- W3, 6, 12, 18). (c, d, e) Changes through time in the different viral categories. The treatment group was further divided in responders (n=3) or non-responders (n=3) based on treatment outcome. Significance was determined through a mixed effect model to account for repeated measures and missing data points followed by Sidak’s multiple comparison test. Asterisks show adjusted *p*-values: * <0.05, **<0.005, ***<0.0005

**Figure 2.**
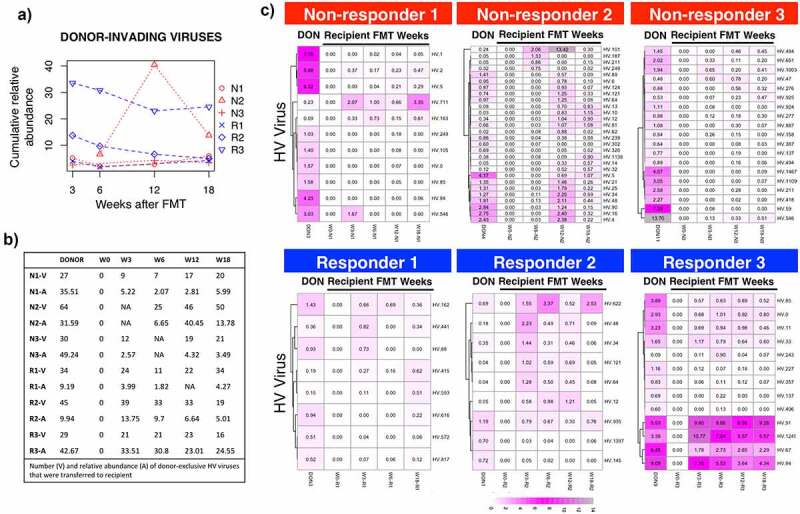
Transfer and establishment of donor-invading viruses after FMT in the recipients gut viral community. (a) Cumulative relative abundance occupied by donor-invading bacteriophages. (b) Number of viruses (v) and their cumulative relative abundance (a) found in donors and recipients at each time point before and after FMT. C) Heatmaps of invading HV groups that take more than 0.05% of the community in at least one time point

In order to gain insights into the impact of FMT treatment on the recipient’s original viral community structure, regardless of taxonomic classification, we investigated the transfer and establishment of bacteriophages before and after the fecal microbial transplant (Figures 1, 2). Conceptually, the viral community of treated MetS subjects before FMT treatment (pre-FMT or baseline) includes viruses that are present both in the healthy donor and in the recipient before treatment (“shared viruses with healthy donor pre-FMT”), and viruses that are only detected in the MetS recipient (“viruses unique to MetS subjects pre-FMT”) (green and pink category, respectively, in Figure 1a top panel). In the case of the controls, since they received their own stool samples, all the viruses found pre-FMT are viruses unique to MetS recipients (pink category in Figure 1a bottom panel).

After FMT treatment, the original community in the recipient will undergo changes that include: (i) changes in abundance profile of viruses found pre-FMT; (ii) in the treatment group only, introduction of “invading viruses” (viruses found in healthy donors pre-FMT, and in their respective recipients only after FMT); (iii) and appearance of newly-activated prophages or viruses that were present below the detection threshold in either the recipients or the donors (“new viruses”). Hence, in subjects from the treatment group, it is not possible to determine whether they come from the donor or from the recipient.

One of the main goals was to examine whether receiving a FMT treatment from a healthy donor could displace viruses found in MetS recipients pre-FMT that do not share any similarities with viruses from their respective healthy donors (“viruses unique to MetS subjects pre-FMT”- pink group in Figure 1). To determine whether this goal was accomplished, the cumulative relative abundance of the different viral categories, before and after FMT, was calculated (Figure 1b–e). In the control subjects that received their own stool samples, the virus community present just prior to FMT was similar to the virus community post FMT treatment (average cumulative abundance of “viruses unique to MetS subjects pre-FMT” W3-W18: 80%±4.6, Figure 1b,c). In contrast, in individuals who received stool treatment from healthy donors only 35%±6.9 of the viral community was maintained between pre- and post FMT treatment (average cumulative abundance of “viruses unique to MetS subjects pre-FMT” W3-W18: 35%±6.9, Figure 1b,c). Overall, this data suggests that transplantation of an allogenic stool sample from a healthy donor significantly reduces the abundance of viruses unique to MetS subjects as compared to receiving a self-stool transplantation (mixed-effect model *p*-value<0.0001, Figure 1c). To investigate whether changes in the control group were greater than the expected variation of the virome over time, a similar analysis was performed on two untreated healthy individuals previously studied in our laboratory.^28^ In these individuals, the viral community found in our initial timepoint was highly maintained 12 and 60 weeks later (98% and 95% cumulative abundance at W12 and W60, respectively). This result is consistent with the literature and highlights the high stability of the gut viral community overtime. It also suggests that the temporal changes observed in the control group are slightly higher than those expected due to temporal variation of the gut viral community (80%±4.6 vs 95–98%). However, future studies should include a no-FMT group within the trial to serve as an internal control for temporal variation. Overall, these results suggest that FMT transplant treatment with a stool sample from a healthy individual significantly reduces the presence of the recipient’s viruses that are not highly related to viruses from the donor’s viral community.

We investigated whether the reduction in “viruses unique to MetS subjects pre-FMT” in the treatment group was due to an increase in new viruses or an increase in donor-like viruses (Figure 1d,e). Analysis of the cumulative relative abundance of both categories suggests that there is a significant increase in new viruses after stool transplantation (25%±5.76 of the post-FMT viral community on average). This increase was significantly higher in the treatment group three weeks after the transplant (10% vs 25% in control and treatment group, respectively; W3 adjusted *p*-value 0.034) (Figure 1d). This result suggests that in the treatment group viruses unique to MetS subjects pre-FMT were replaced by new viruses that were not originally present in either the healthy donors or control subjects at detectable abundances.

**Figure 3.**
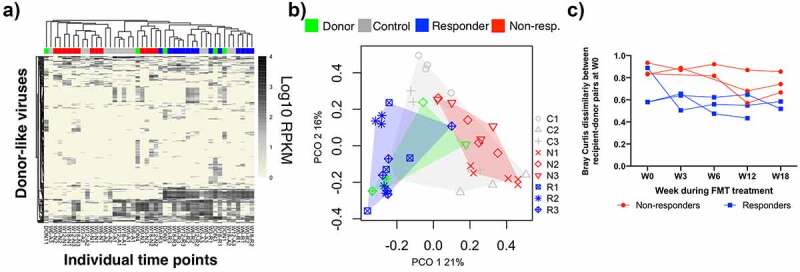
Analysis of donor-like HV viruses dynamics during the FMT time course. (a) Heatmap representation of log10 raw abundance of the 232 HV viruses that were shared between at least one healthy-donor recipient pair. Columns are organized based on average hierarchical clustering of Bray-Curtis dissimilarity and rows are organized based on complete hierarchical clustering of Euclidean distance. (b) Principal Coordinate Analysis of Bray-Curtis dissimilarity index of raw abundances of shared HV groups. Points represent individual time points. Number on top of the point represents week within the FMT course. (c) Bray-Curtis dissimilarities between healthy donors and their respective recipients

Next, we investigated whether there are differences in the donor-like viral community within subjects in the treatment group before and after treatment (Figures 1, 2, 3). First, we determined whether there were differences in the cumulative relative abundance of donor-like viruses (summation of relative abundance of donor-like bacteriophages). On average, 24%±8.7 of the community was taken by donor-like bacteriophages before FMT treatment (Figure 1e). After the FMT treatment, the average increased slightly (37%±6.4, ns) and remained fairly stable within most individuals. No statistical differences were observed between treatment groups. We investigated the dynamics of donor-invading viruses and their ability to get established within the recipients (Figure 2). On average, 38 ± 14 donor-invading viruses (HV groups) could be detected in at least one time point after the FMT treatment (Figure 2a,b). There were no significant differences in the number or abundance of invading donor phage between treatment groups. It was noticeable that Responder 3 had a very low percentage of donor-like bacteriophages pre-FMT but that after treatment multiple donor-invading phage were established throughout the rest of the sampling time course (Figure 2a,c bottom right panel). With the exception of Responder 3 and Responder 1, there was no correlation between the relative abundance of donor invading phage originally in the donor compared to their relative abundance once established in the recipient (mean R^2^ adjusted = 0.18 ± 0.27). Interestingly, Non-responder 2 showed a spike in donor-invading contigs 12 weeks after FMT treatment. Some of these viruses were classified as *Siphoviridae* bacteriophages, suggesting that an unknown event could have led to an activation of prophages or latent viruses. Overall, these results show that the cumulative relative abundances of donor-like viruses were not different among FMT treatment groups and that only a small percentage of donor-invading viruses was found in MetS subjects after FMT treatment.

We explored whether the donor viral community profile could inform us about differences between treatment subgroups (Figure 3). We began by looking for viruses that were shared between a healthy donor and its respective MetS recipient. There were 232 HV groups shared by at least one pair (Figure 3a). Some of these HV groups were also found in MetS individuals that received a non-treatment FMT (controls), hence they were included in subsequent analysis. Principal Coordinate Analysis (PCO) was applied to Bray-Curtis distance matrix between treatment subjects and their donors at every time point (Figure 3b). Responders’ samples clustered significantly away from those of the non-responders (PERMANOVA centroid analysis R-N *p* = .003, Table S3). Interestingly, contrary to non-responders, the centroid distance between responders and donors was not significantly different (N-D *p* = .003, R-D *p* = .354, Table S3). For clarity, the particular dissimilarities between each healthy donor-MetS recipient pair in the treatment group were separately graphed (Figure 3c). With the exception of Responder 3, all non-responders were very dissimilar to their donors through time. Importantly, Responder 3 dissimilarity with its donor dropped sharply in the first weeks after treatment. Within the non-responder group, dissimilarities with the donor remained high up to six weeks post-treatment. Overall, these results indicate that even if responders and non-responders have a similar fraction of their community occupied by donor-like viruses, their relative abundance profile is more similar between responders and their donors than between non-responders and their donors.

### Identification of phage groups enriched within treatment groups and their relationship with clinical outcome measures

**Figure 4.**
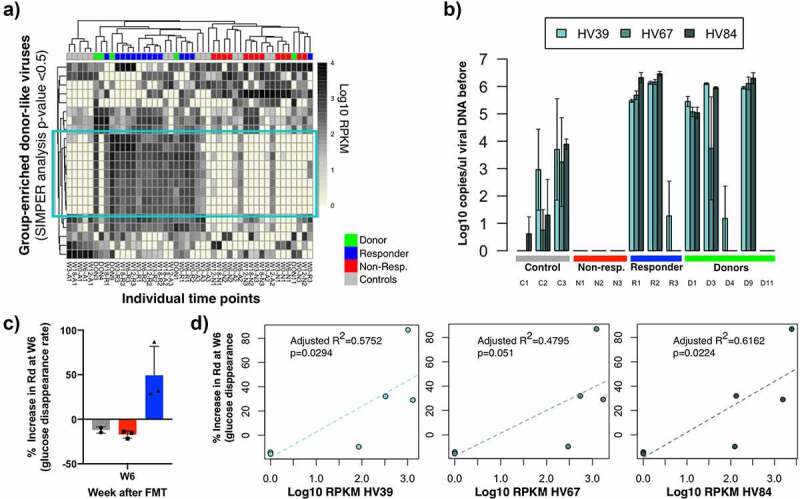
Identification of phage groups enriched in treatment outcome subgroups. (a) Heatmap representation of log10 raw abundance of the HV viruses that significantly contribute to separate treatment groups in the ordination space in Figure 4b (simper analysis with 1000 permutations, *p*-value <0.05). Columns are organized based on average hierarchical clustering of Bray-Curtis dissimilarity and rows are organized based on complete hierarchical clustering of Euclidean distance. (b) Detection of these HV groups in study subjects pre-FMT using a q-PCR based assay. C) Percentage increase in glucose disappearance rate (Rd) from study subjects in this pilot study. The value of one control individual was removed (See statistical analysis). D) Correlation analysis between phage enriched in responder groups and the increase in Rd rate in subjects six weeks after transplant

Since there were differences between treatment subgroups (responders vs non-responders), we sought to identify specific bacteriophages enriched in these subgroups (Figure 4). To identify phage groups that explained most of the variation within the ordination analysis we applied similarity percentages analysis (SIMPER). From the 232 donor-like HV groups used for the PCO analysis (Figure 3b), there were 22 HV groups that contributed significantly to separate treatment groups (simper permutation analysis *p*-value<0.05, Figure 4a). From these, there was a subset of 10 HV groups that were mostly absent in non-responders pre-FMT but were found at high levels in responders. The only responder that did not harbor these HV groups pre-FMT (Responder 3), acquired and maintained them after FMT treatment. None of the non-responders acquired these 10 HV groups. Five of these groups were previously classified as Caudovirales (lowest rank classification). To confirm the presence and absence of these Caudovirales HV groups, we performed q-PCR based analysis of three of these HV groups 39, 67, 84 (Figure 4b, See Material and Methods). The three groups were detected in responders and their donors pre-FMT (Figure 4b) and through-out the FMT time course. The same viral sequences were not detected in any of the viral DNA from the 3 non-responders.

Next we determined whether there was any correlation between the abundance of the 3 tested HV bacteriophages with clinical outcome measures. In the original FMT trial, subjects within the treatment group were separated in responders and non-responders based on the increase in glucose disappearance rate (% increase Rd) (Figure 4c) ^14^ A correlation analysis between the abundance of HV virus 39, 67, and 84 and the % increase in Rd in individuals from the control and treatment group was performed. Specifically, we tested whether there was a relationship between these two variables at six weeks post-FMT treatment (W6) because in the original trial differences in the %Rd increase between responders and non-responders were only significant up to six weeks after FMT.^14^ This analysis revealed that the abundance of HV39 and 84 was significantly correlated with the percentage increase in Rd at six weeks post-FMT (*p*-value <0.05, adjusted R^2^ > 0.5 Figure 4d). There was a small but not significant correlation between HV67 and this clinical outcome (*p*-value = 0.051, R^2^ = 0.4795). These results suggest that within the limits of this small pilot study we have identified 2 bacteriophage groups that are associated with a relevant clinical outcome.

### Prevalence of bacteriophages enriched in treatment outcome groups among a larger set of MetS subjects before FMT treatment

**Figure 5.**
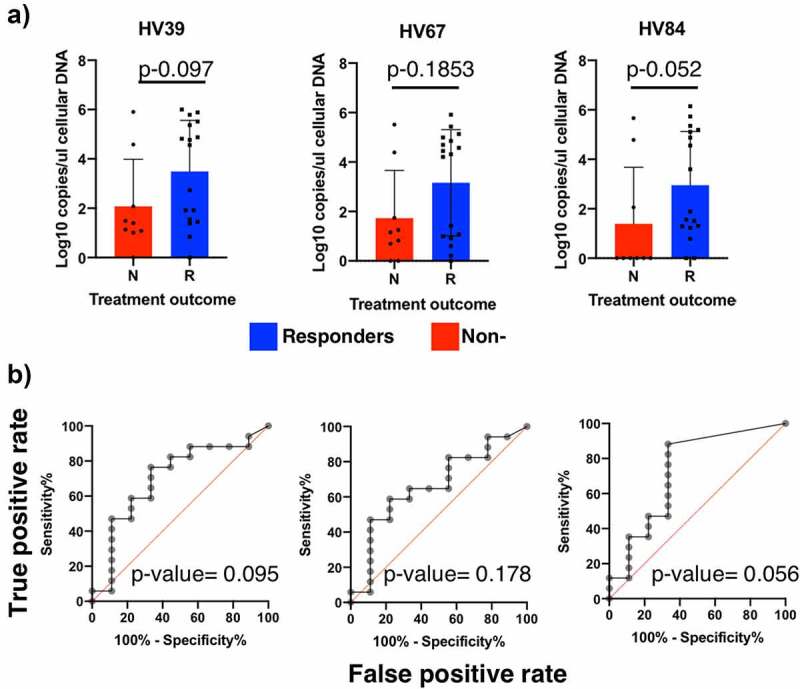
Prevalence of bacteriophages enriched in responder subjects in this study among a larger set of MetS subjects before FMT treatment. (a) HV abundance in cellular DNA extracted from stool samples of treatment subjects in Koote et al. FMT trial determined through q-PCR analysis. (b) ROC curves analysis of HV abundances pre-FMT. The area under the curve and its correspondent *p*-value determine the suitability for these HV groups to be used as predictors for treatment outcome

Next, we wanted to determine the prevalence of these HV groups (HV39, 67, and 84) pre-FMT in a larger set of MetS subjects that had undergone FMT treatment (Figure 5a). To do this, we performed a q-PCR analysis in the 38 subjects from the FMT trial under study. This includes the 9 subjects used to initially identify the enriched HV groups, and 29 additional subjects (total n = 38).^14^ Previous results had shown that of the 26 subjects that had received FMT treatment from a healthy donor, 17 of these subjects had shown clinical improvement after the FMT (65%), while the remaining 9 subjects did not show clinical improvement (35%).^14^ Testing of stool samples from these 26 subjects for the presence of these 3 bacteriophages revealed that 12 subjects contained high levels of at least two of the three bacteriophages (average 2 × 10^5^ copies/μl) (Figure 5a). Consistently with what we found in our smaller subgroup, the majority of individuals that had high levels of these phage groups presented a clinical improvement (83% (10 true responders/12 subjects with high HV levels). In the initial treatment subjects that we examined, 100% of the subjects were devoid or contained low levels of these HV groups did not respond to the treatment. In contrast, the screening of the larger set of MetS subjects revealed that approximately 50% of the subjects that were devoid or contained low levels of these HV groups did respond to treatment (7 true responders/14 subjects with low HV levels). We did not determine whether these individuals acquired these HV groups immediately after FMT, similarly to Responder 3 in this study.

Receiver Operating Characteristic (ROC) analysis revealed that none of the HV groups were a significant discriminant factor in predicting treatment outcome (area under the curve *p*-value >0.05), even though HV-84, which had the highest adjusted R^2^ values in the Rd linear models, was almost significant (*p*-value = 0.0557) (Figure 5b). Combining the abundance of these three HV groups was not significant either. This is likely due to the high false-negative rate obtained in a larger MetS cohort. Overall, these results suggest that even though almost all subjects that presented high levels of these HV groups presented clinical improvement, the sensitivity of this assay is insufficient to use them as predictors for treatment outcome.

## Discussion

The overall objective of this study was to investigate changes in the gut bacteriophage community in MetS subjects that undergo FMT treatment in a small pilot study. Our results indicate that the gut viral community undergoes significant changes after receiving FMT treatment (Figure 1). Interestingly, we identified differences between the viral communities present in responders vs. non-responders. In particular, we found that the dissimilarity between the viral community shared by recipients and their respective donors was greater in subjects that did not respond to treatment (either before or in the first weeks after FMT) (Figure 3). Within the virus groups that could explain most of the dissimilarities, we identified phage groups that were associated with the main clinical outcome variable (percentage increase in glucose disappearance rate- Rd) (Figure 4). We investigated the prevalence of these HV phages in a larger set of MetS subjects that had undergone FMT treatment. We found that even though the majority of individuals that had high levels of these HV phages did show clinical improvement, these phage groups were not discriminant enough to use them as biomarkers for predicting treatment outcome (ROC *p*-value: 0.09, 0.178, 0.056). Overall, these results are promising and should provide support for a more powered expanded FMT study.

Interest in the role of bacteriophages in the gut microbial community has increased.^22,30^ Evidence for the beneficial impact of gut bacteriophages in human health is emerging.^20^ We previously reported the existence of a healthy gut phageome (HGP) in healthy individuals that was less prevalent in subjects with inflammatory bowel disease (IBD).^20^ We did not find similar decreased prevalence of the HGP in MetS subjects, suggesting that changes in the HGP might be only characteristic of certain gastrointestinal diseases. Even though the beneficial symptoms of FMT treatment are generally attributed to the bacterial component of the transferred stool, it is likely that, in conjunction with bacteria, additional members of the microbial community such as bacteriophages or fungi contribute to clinical improvement in treated subjects.^31^ In a recent pilot study, transplantation of fecal filtrates (FFT) alone restored health in CDI patients.^32^ Fecal filtrates are highly enriched in bacteriophages, suggesting that FFT benefits may be at least partially attributed to bacteriophages. Interestingly, a recent study showed that certain viruses encode insulin-like peptides, which are sufficient to stimulate insulin signaling, suggesting that specific viral features could contribute to health in MetS subjects.^33^ However, it is important to note that FFT can also induce disease symptoms, emphasizing the importance of a balanced phage community to maintain health.^34^

Analysis of the role of bacteriophages during FMT in other disease subjects has been reported (i.e. CDI and ulcerative colitis-UC).^24,35–38^ Zuo et al. reported that bacteriophages could influence the outcome of CDI-FMT.^24^ In this study, individuals with CDI presented lower Caudovirales-richness than healthy donors at pre-FMT. Following FMT treatment, a high relative abundance of healthy donors’ invading phage was significantly associated with treatment success.^24^ In patients with UC, differences in richness of the bacteriophage community before or after the transplant were not associated with treatment outcome.^38^ However, more specific differences were not investigated.^38^

In our study, there were not significant differences in bacteriophage community richness or diversity between treatment groups (healthy donors, MetS controls, MetS that received healthy donor FMT). Differences in total Caudovirales abundance between responders and non-responders were not detected either, and viruses from the *Microviridae* family were underrepresented. Additionally, the healthy donors’ invading phage profile was similar between treatment subgroups (responders vs non-responders), and no specific patterns of bacteriophage transfer based on viral family classification were identified.^37^ Instead, responders could be differentiated from non-responders based on the community structure, and the phage community of responders and donors had a greater overlap (Figure 3). Therefore, we hypothesized that MetS subjects with a less distorted phage community at pre-FMT are more likely to show clinical improvement. These differences likely arise from differences in disease etiology, suggesting that evidence of the impact of bacteriophages in a specific disease cannot be extrapolated to other health conditions.

Changes in the phage community of recipients after recipients might arise from two different sources (Figure 1): (1) establishment of donor invading phages, and (2) changes in the recipient’s phage community profile. Invading phages can be extracellular donor phages that successfully find and infect a host in the recipient’s established microbial community or donor prophages that are transferred inside their ‘Trojan bacterial-horse’ (phage integrated in the bacterial host genome).^37^ Similar to donor invading bacterial strains, donor invading phages can be transferred and maintained through time in the recipient, but typically constitute a small percentage of the post-FMT phage community.^39^ Changes in the recipient’s phage community that is successfully established will be primarily due to activation of previously inactive recipient prophages, as well as an increase in the relative abundance of pre-FMT low-abundance phages. The biggest changes were seen in the profile of the bacteriophages that were unique to the MetS recipient, particularly when they received a healthy donor stool sample, suggesting that the new community and the donor-community are able to displace these types of bacteriophages. Future studies directed at the mechanism(s) of phage establishment and the direct function that phage play during FMT treatment in shaping the function and structure of gut microbial community are warranted.

The main limitation of our study is the small sample size per group. Despite this limitation, we were able to determine that FMT treatment, in absence of previous antibiotic treatment, significantly changes the bacteriophage community of the recipients. However, changing the bacteriophage community is not sufficient to present a clinical improvement in all the individuals. This is not surprising, since there is evidence of an association between the bacteriophage community structure and certain diseases,^40^ but a causal effect remains to be proven. It will be important to determine the magnitude and specific features of the changes that are necessary to present a clinical improvement in different diseases. In the case of MetS syndrome, results from this pilot study should be validated in a larger number of subjects in order to unequivocally demonstrate that the bacteriophage community is associated with treatment outcome. Additionally, this study does not address bacteriophage-host dynamics. Differences in the interaction between bacteriophages and their host, for example, due to host availability, could explain why individuals such as Responder 3 sustained a more dramatic and successful establishment of donor-invading bacteriophages shortly after the FMT treatment. Whole-genome sequencing of both, the bacterial and bacteriophage community will shed light into phage-host dynamics during FMT treatment. An extended analysis could also be useful to identify more robust biomarkers suitable for predicting clinical outcomes. Finally, analysis of the compatibility between donor and recipient and its influence on the treatment outcome using a greater number of FMT pairs could be highly informative in FMT clinical trial design.

## Material and methods

Extended material and methods are available in supplemental data.

### Sample source and selection

The FMT trial herein analyzed is described in detail in Kootte et al.^14^ In brief, male, omnivorous, Caucasian, obese subjects with MetS were enrolled in a FMT clinical trial to test the efficacy of FMT treatment to treat MetS (see Table S1 from Kootte et al. for all subjects’ characteristics^14^). In this trial, subjects were randomly assigned to the control treatment group (self-stool microbial transplant) and treatment (healthy donor stool microbial transplant) treatment group. The control group informs of the dynamics associated with the fecal transplant procedure alone (bowel lavage followed by stool microbial community transplant), regardless of the stool microbial community source. A portion of each of the individual fecal samples was frozen for analysis of the microbial and bacteriophage community. The remaining sample content was prepared for transplantation by homogenization in 500cc of 0.9% sterile saline and filtration through a metal sieved. Within 6 hours of fecal sample preparation, subjects received a bowel lavage, followed by the transfer of the processed stool. A key clinical outcome measure was the rate of glucose disappearance (Rd rate), which is a measure of insulin sensitivity. For the small pilot study described here, a subset of these subjects was selected to analyze the bacteriophage community dynamics. Three individuals from the control treatment group were randomly selected (see Table S1 for subjects’ characteristics in this study). Additionally, six individuals from the treatment group, three with the highest and three with lowest Rd rate were selected as responders and non-responders, respectively.

### Virus purification and sequencing data process

Fecal samples (1 g) were resuspended in SM buffer, span down and subsequently filtered through 2 and 8-micron filters (fecal filtrates) (See extended material and methods in supplementary data). After DNAse treatment, DNA from viral fecal filtrates was extracted and the DNA was sequenced using HiSeq Illumina technology (Table S2). Reads were quality trimmed with trimmomatics [33] and assembled with IDBA.^41^ Reads from individual study subjects were pooled together and assembled. It is important to note that metagenomes from different individuals were not pooled together at any time. In total, 14 assemblies were generated: 9 cross-assemblies for subjects (3x autologous, 3x non-responders and 3x responders FMTs) and 5 single-subject assemblies for each healthy donor. High-quality reads were de-duplicated. To normalize for different sequencing depths between samples, reads from each metagenome were randomly subsampled at a constant depth of 1,367,462 reads per time point and these subsampled reads were used to mapped to the assembled contigs. Contig abundance was determined based on number of Reads Per (contig) Kb per Million reads (RPKM). Contigs with hits to 16S rDNA were eliminated. Efforts in removing bacterial contamination were discrete to avoid removing prophage sequences. Only contigs with >7RPKM (minimum RPKM in donors) were considered present.

### Homologous virus (HV) identification

Assembled contigs > 3000 bp were used to determine HV groups. Specifically, an all-to-all BLASTn analysis was performed using these contigs and default parameters, except e-value was set to 1e-20 and hsp-to-query-ratio was 0.5.^42,43^ The resulting BLASTn file was parsed using network analysis to determine group membership.^28,44^

### Taxonomic classification

ORFs were predicted with prodigal^45^ for all individual viral contigs (n = 28,869) and compared to the POG database.^28,29^ Proteins longer than 100aa with less than 1% of their length in ambiguities were queried against the POGs database (PSI-BLAST default values except e-value set as <1e-5). Contigs that contain at least one taxa marker gene were classified based on Waller et al.^28,29^ When multiple sequences contained a taxa marker gene and were in the same HV group, their taxonomy was always concordant, therefore, their taxonomy was extended to all contigs from the group.

### Ordination analysis

Bray-Curtis dissimilarity matrix was calculated using the Vegan R package,^46^ and PCO was carried out using LabDSV^47^ and the Vegan R package^46^ respectively. Analysis of centroids was performed through PERmutational Multivariate ANalysis Of VAriance (PERMANOVA) using the Adonis function within the Vegan R package.^48^

### Identification of HV groups that contribute to treatment outcome differences identification and analysis

HV groups that significantly separated responders from non-responders were identified using the dissimilarity index generated with the SIMPER function from the vegan package in R, based on treatment group (donor, controls, non-responders and responders), using 1000 permutations.^46^ HV groups that separated treatment groups with a cumulative sum <0.7 and a *p*-value < 0.05 were selected.

### qPCR analysis selected HV group

HV groups that separated treatment groups with a cumulative sum <0.7 and a *p*-value < 0.05 after SIMPER analysis, that were missing in the Non-responder groups (square in Figure 5a) and that were classified as Caudovirales were selected for further analysis (HV groups 39, 67, 84, 85 and 0). Because HV groups encompass at least 2 phage sequences (contigs) or more, individual contigs these HV groups (n-contigs = 1067) were extracted and assembled together within each HV group into a total of 425 contigs (Geneious assembly custom sensitivity settings; 1500 bp at 90% identity).^49^ Reads were re-mapped to these contigs using bowtie2 and normalized based on RPKM (see sequencing data processing). Contigs were scored based on abundance and presence in responders, non-responders and donors to identify sequences suitable for qPCR analysis. First, the total RPKM per contig in all responders and donors, and separately in non-responders was calculated. Then, it was multiplied, respectively, by the number of responders and donors (R-score) or non-responders (N-score) that had that contig. Subsequently, the R-score was divided by the N-score plus 1 and multiplied by the median number of reads in responders and donors, rendering the Score for each contig. The highest-scored contigs per HV group were visually inspected. Primers to conserved areas between subjects, POG genes when possible, were designed using Geneious.^49^ Target sequences were amplified using q-PCR, both in total cellular DNA and in viral filtrate DNA. qPCR on viral DNA was done in 1:100 dilution of DNA material in triplicate using SsoAdvanced™ Universal SYBR® Green Supermix. qPCR on cellular DNA was done in 20 ng of DNA material in triplicate SsoAdvanced™ Universal SYBR® Green Supermix. Copies per ul were determined using standard curves.

### Statistical analysis

Differences in viral diversity and richness pre-FMT and through time were determined using t-test and mixed effect model analysis to account for repeated measures and missing data points followed by Sidak’s multiple comparison test using PRISM software. Differences in the cumulative relative abundances of different viral community groups (e.g. “viruses unique to MetS subjects pre-FMT”) was determined using a mixed effect model analysis to account for repeated measures and missing data points using PRISM software. Analysis of centroids after ordination analysis was performed through permutation analysis using Adonis within the Vegan R package.^48^ Linear models of the HV abundance and Rd rate were performed in using the lm function in R. For this analysis, control subject 1 was removed because its % increase in Rd value was a great outlier even though its Rd values were lower than almost all subjects in the trial (See Figure 2 in Kootte et al. 2017^14^). Statistical differences in the HV abundance after q-PCR analysis were determined using the non-parametric Mann–Whitney test in PRISM software. ROC curve analysis was performed using PRISM software.

## Supplementary Material

Supplemental Material

## Data Availability

Sequencing data available upon publication at NCBI under the following BioProject number: PRJNA499081 (http://www.ncbi.nlm.nih.gov/bioproject/499081).
