# Supplementary material for: Gut bacteriophage dynamics during fecal microbial transplantation in subjects with metabolic syndrome
Source: Gut Microbes. 2021 Apr 1;13(1):1897217. doi: 10.1080/19490976.2021.1897217 (PMC8023239; doi:10.1080/19490976.2021.1897217)
Supplement: Supplemental Material [file KGMI_A_1897217_SM5623.docx]

# Supplemental Material and Methods

## Virus purification

One gr of fecal sample was resuspended in 3 ml of SM buffer, thawed on ice for 10 min, vortexed for 1 min at 3,000rpm and left on ice for 2 hours. Sample was span at 4,200g for 45 min at 4C to remove bacterial contamination. Supernatant was recovered and filtered through a pre-washed 2-micron filter ((Millipore AP2504200). Filtrate was span again at 5,000g for 45 min at 4C. Supernatant was filtered through a 0.8-micron filter (Pall corporation Acrodisc syringae filters 4618). CsCl purification was not used to avoid introducing bias against certain viral types. ^1^ Efforts focused on DNA viruses since most RNA viruses in the gut are plant viruses. ^2^ Five hundred microliters of viral filtrate were treated with DNAse (RQ1 Promega) and DNA was subsequently extracted using PureLink® Viral RNA/DNA Mini Kit (Thermo Fisher, 12280050).

## Sequencing data processing

Viral filtrates’ DNA were sequenced with HiSeq Illumina technology. A total of 251,330,194 paired-end sequencing reads were obtained (250bp) (Table S1). Reads were quality trimmed with trimmomatics.^3^ Reads belonging to one individual were pulled together (cross-assembly) and assembled with IDBA (minkmer=100, maxkmer=250). ^4^ Reads were pooled based on individual source (all time points from one individual) and assembled. In total, 14 assemblies were generated: 9 cross-assemblies for patients (3x autologous, 3x non-responders and 3x responders FMTs) and 5 single-assemblies for each healthy donor. Individual high-quality reads were de-duplicated, subsampled (1,367,462 reads per time point) and mapped back to their respective assembled contigs (81% of subsampled reads were mapped). Contig abundance was determined based on number of Reads Per (contig) Kb per Million reads (RPKM). Contigs with hits to 16S were eliminated (30,389 contigs remained, >99%). Because the gut prophage content is underrepresented in current databases, gut prophages are hard to predict based on hits to known bacteriophage sequences. Thus, our efforts in removing bacterial contamination are discrete to avoid removing real prophage sequences from our data set. Only contigs that had an abundance >7RPKM (minimum RPKM in donors) were considered present and focus of further analysis (28,869 contigs).

## Homologous Virus Identification

All-to-all BLASTn analysis was performed with default parameters, except e-value cutoff, which was set to 1E-20, max target sequences to 10,000, and 0.5 hsp_to_query_ratio.^5, 6^ BLASTn file was parsed using network analysis to determine sequence membership. ^7, 8^

## Taxonomic classification

ORFs were predicted with prodigal ^9^ for all individual sequences (n=28,869) and compared to the POG genes database as described in Manrique et al 2016. ^8, 10^ The contigs that contain a taxa marker gene were classified (n=2,278; 798 singletons and 1,480 sequences belonging to 329 HV groups). When there were multiple sequences with a taxa marker gene in the same HV group, the taxonomy was always concordant, therefore, the taxonomy was extended to all the contigs from the group.

## Ordination analysis

Bray-Curtis dissimilarity index was calculated on the virus abundance matrix of normalized reads (RPKM). Principal coordinate analysis (PCO) was carried out using LabDSV package in R. ^11, 12^ Analysis of centroids was done through permutation analysis with adonis() function from the vegan package in R. ^13^

## Biomarker and qPCR analysis

HV groups that separated treatment groups with a cumulative sum <0.7 and a p-value < 0.05 after SIMPER analysis, that were missing in the Non-responder groups (square in Fig. 5A) and that were classified as Caudovirales were selected for further analysis (HV groups 39, 67, 84, 85 and 0). Because HV groups encompass at least 2 phage sequences (contigs) or more, individual contigs these HV groups (n-contigs=1067) were extracted and assembled together within each HV group into a total of 425 contigs (Geneious assembly custom sensitivity settings; 1500bp at 90% identity). ^14^ Total reads were remapped to this set of contigs with bowtie2 and normalized based on RPKM (see sequencing data processing). Contigs were scored based on abundance and presence in each outcome group and donors. First the total RPKM per contig in all responders and donors, and separately in non-responders was calculated. Then it was multiplied respectively by the number of responders and donors (R-score) or non-responders (N-score) that had that contig. Subsequently, the R-score was divided by the N-score plus 1 and multiplied by the median number of reads in responders and donors, rendering the Score for each contig. The highest-scored contigs in the Caudovirales HV groups were visually inspected. Primers to identified conserved areas between subjects were designed. Target sequences were amplified using q-PCR, both in total DNA and in viral filtrate DNA. PCR on viral DNA (Figure 5A) was done in 1:100 dilution of DNA material in triplicate using SsoAdvanced™ Universal SYBR® Green Supermix (cat #1725274). qPCR on cellular DNA (Figure 6A) was done in 20ng of DNA material in triplicate SsoAdvanced™ Universal SYBR® Green Supermix.

# Supplemental Figures

## Figure S1. Experimental design





## Figure S2. Viral diversity before and after FMT treatment


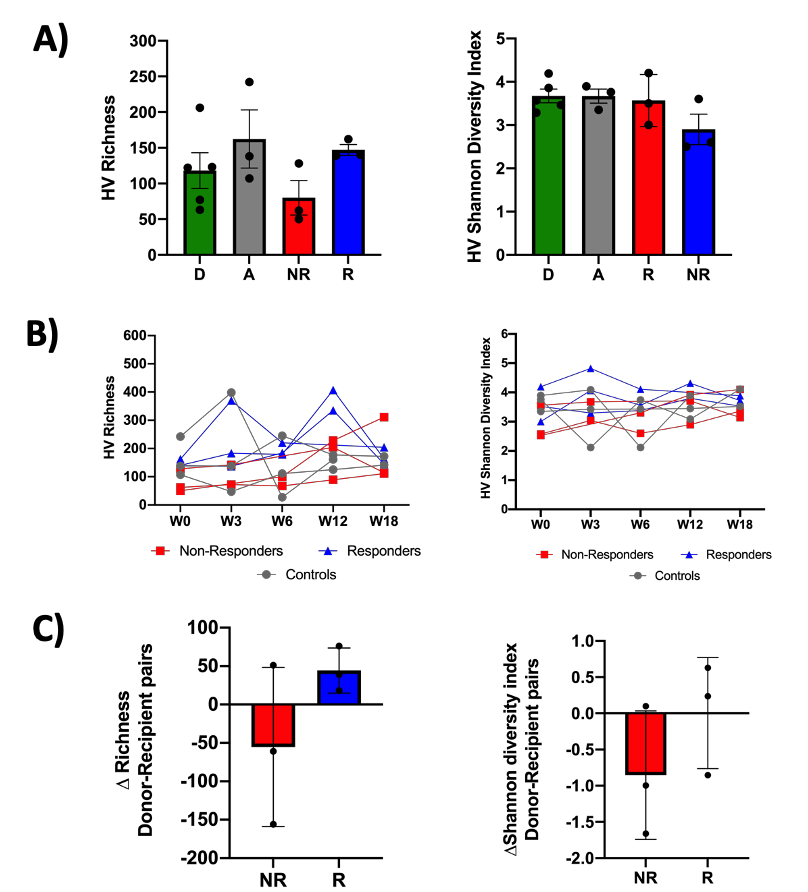


## Table S1. Baseline characteristics of study subjects

|  | **NR (n=3)** | **R (n=3)** | **C (n=3)** | **D (n=3)** | **Ideal range** |
| --- | --- | --- | --- | --- | --- |
| **Male gender (%)** | 100% | 100% | 100% | 100% | NA |
| **Age (years)** | 60±5 | 59±5 | 50 ±3 | 25±3 | NA |
| **Weight (kg)** | 123±17 | 107±12 | 127±16 | 72±8 | NA |
| **BMI (kg/m2)** | 34±3 | 33±3 | 39±8 | 22±1 | 18.5-24.9 |
| **Waist circumference (cm)** | 119+2 | 121±3 | 122±2 | 83±3 | <102 |
| **Blood pressure: systolic (mmHg)** | 152±8 | 145±20 | 138±12 | 118±3 | <120 |
| **Blood pressure: diastolic (mmHg)** | 100±6 | 85±10 | 88±8 | 74±4 | <80 |
| **Fasting glucose (mmol/L)** | 6.2±0.5 | 6.8 ±0.5 | 6.6±0.7 | 5.1±0.4 | <6.1 |
| ***Insulin (pmol/L)*** | 133±52 | 123±12 | 85±21 | 20±9 | <174 |
| **HOMA-IR (Insulin Resistance)** | 5.1±1.8 | 5.3 | 3.7±0.6 | 0.7±0.2 | <1 |
| **Cholesterol total (mmol/L)** | 6.1±0.6 | 5.5±0.4 | 5.4±0.9 | 4.8±0.9 | <5.2 |
| **HDL cholesterol(mmol/L)** | 1.2±0.1 | 1.2±0.1 | 1.1±0.3 | 1.5±0.3 | > 1 |
| **LDL cholesterol (mmol/L)** | 4.3±0.6 | 3.7±0.3 | 3.3±0.5 | 2.9±0.9 | <3.4 |
| **Triglycerides (mmol/L)** | 1.2±0.1 | 1.3±0.7 | 1.9±0.5 | 0.8±0.3 | < 1.7 |
| NR: Non-responder, R: Responder; C: Control; D: Donor | | | | | |

## Table S2. Sequencing reads information

| **TOTAL READS** | **Total reads** | **Trim-paired reads** | **Trim-paired unique** |
| --- | --- | --- | --- |
| **Autologous 1** | 28,939,622 | 27,717,648 | 10,525,467 |
| **Autologous 2** | 29,374,784 | 27,460,216 | 10,540,996 |
| **Autologous 3** | 19,409,994 | 18,591,030 | 8,924,706 |
| **Non-responder 1** | 28,707,404 | 27,248,156 | 8,606,954 |
| **Non-responder 2** | 24,305,938 | 22,977,676 | 8,308,225 |
| **Non-responder 3** | 21,627,458 | 20,575,494 | 11,971,200 |
| **Responder 3** | 23,927,384 | 22,981,844 | 11,164,515 |
| **Responder 1** | 21,713,756 | 20,672,964 | 11,863,007 |
| **Responder 2** | 28,313,130 | 27,024,138 | 13,387,816 |
| **Donor1** | 4,980,824 | 4,799,670 | 1,953,526 |
| **Donor3** | 5,106,248 | 4,935,244 | 3,168,536 |
| **Donor4** | 5,137,418 | 4,926,610 | 3,478,522 |
| **Donor9** | 4,794,106 | 4,671,770 | 1,483,940 |
| **Donor11** | 4,992,128 | 4,825,082 | 1,463,399 |
| **TOTAL** | 251,330,194 | 239,407,542 | 106,840,809 |

## Table S3. PERMANOVA centroid analysis of donor-like viruses (Fig. 4B)

| **ADONIS** | **Df** | **Sum of Squares** | **Mean of Squares** | **F.Model** | **R2** | **Pr(>F)** |
| --- | --- | --- | --- | --- | --- | --- |
| met.fmt$Group | 3 | 3.336 | 1.112 | 4.257 | 0.233 | 0.001*** |
| Residuals | 42 | 10.972 | 0.261 |  | 0.767 |  |
| Total | 45 | 14.308 |  |  | 1.000 |  |
| adonis(formula = fmt.hdvi[, colnames(donor.like)] ~ met.fmt$Group) | | | | | | |
| Permutation number=999 | | | | | | |

| **PAIRWISE ADONIS** | **F Model** | **R2** | **p.value** | **Adjusted p.value** | **sig** |  |
| --- | --- | --- | --- | --- | --- | --- |
| Donor vs Controls | 2.453 | 0.126 | 0.009 | 0.054 |  |  |
| Donor vs Nonresponders | 3.240 | 0.168 | 0.003 | 0.018 | . |  |
| Donor vs Responder | 1.584 | 0.085 | 0.117 | 0.702 |  |  |
| Controls vs Nonsresponders | 3.924 | 0.136 | 0.001 | 0.006 | * |  |
| Controls vs Responders | 5.454 | 0.173 | 0.001 | 0.006 | * |  |
| Nonresponder vs Responders | 7.726 | 0.236 | 0.001 | 0.006 | * |  |
| pairwise.adonis(x = fmt.hdvi[,colnames(donor.like)], factors = met.fmt$Group, sim.method = 'bray', p.adjust.m = 'bonferroni') | | | | | |  |
|  |  |  |  |  |  |  |

# REFERENCES

1. Kleiner M, Hooper LV, Duerkop BA. Evaluation of methods to purify virus-like particles for metagenomic sequencing of intestinal viromes. BMC Genomics 2015; 16:7. doi: 10.1186/s12864-014-1207-4

2. Zhang T, Breitbart M, Lee WH, Run JQ, Wei CL, Soh SW, Hibberd ML, Liu ET, Rohwer F, Ruan Y. RNA viral community in human feces: prevalence of plant pathogenic viruses. PLoS Biol 2006; 4:e3. doi: 10.1371/journal.pbio.0040003

3. Bolger AM, Lohse M, Usadel B. Trimmomatic: a flexible trimmer for Illumina sequence data. Bioinformatics 2014; 30:2114-20. doi: 10.1093/bioinformatics/btu170

4. Peng Y, Leung HC, Yiu SM, Chin FY. IDBA-UD: a de novo assembler for single-cell and metagenomic sequencing data with highly uneven depth. Bioinformatics 2012; 28:1420-8. doi: 10.1093/bioinformatics/bts174

5. Santiago-Rodriguez TM, Ly M, Bonilla N, Pride DT. The human urine virome in association with urinary tract infections. Front Microbiol 2015; 6:14. doi: 10.3389/fmicb.2015.00014

6. Santiago-Rodriguez TM, Ly M, Daigneault MC, Brown IH, McDonald JA, Bonilla N, Vercoe EA, Pride DT. Chemostat culture systems support diverse bacteriophage communities from human feces. Microbiome 2015; 3:58. doi: 10.1186/s40168-015-0124-3

7. Bolduc B, Wirth JF, Mazurie A, Young MJ. Viral assemblage composition in Yellowstone acidic hot springs assessed by network analysis. The ISME journal 2015. doi: 10.1038/ismej.2015.28

8. Manrique P, Bolduc B, Walk ST, van der Oost J, de Vos WM, Young MJ. Healthy human gut phageome. Proc Natl Acad Sci U S A 2016; 113:10400-5. doi: 10.1073/pnas.1601060113

9. Hyatt D, Chen GL, Locascio PF, Land ML, Larimer FW, Hauser LJ. Prodigal: prokaryotic gene recognition and translation initiation site identification. BMC Bioinformatics 2010; 11:119. doi: 10.1186/1471-2105-11-119

10. Waller AS, Yamada T, Kristensen DM, Kultima JR, Sunagawa S, Koonin EV, Bork P. Classification and quantification of bacteriophage taxa in human gut metagenomes. The ISME journal 2014; 8:1391-402. doi: 10.1038/ismej.2014.30

11. Dixon P. VEGAN, a package of R functions for community ecology. Journal of Vegetation Science 2003; 14:927-30. doi: doi:10.1111/j.1654-1103.2003.tb02228.x %U <https://onlinelibrary.wiley.com/doi/abs/10.1111/j.1654-1103.2003.tb02228.x>

12. Roberts DW. labdsv: Ordination and multivariate analysis for ecology. 2007. doi:

13. Anderson MJ. Permutational Multivariate Analysis of Variance (PERMANOVA). Wiley StatsRef: Statistics Reference Online.

14. Kearse M, Moir R, Wilson A, Stones-Havas S, Cheung M, Sturrock S, Buxton S, Cooper A, Markowitz S, Duran C, et al. Geneious Basic: an integrated and extendable desktop software platform for the organization and analysis of sequence data. Bioinformatics 2012; 28:1647-9. doi: 10.1093/bioinformatics/bts199
